# It’s all a matter of perspective—a comparison of scientific experts’ vs. farmers’ opinions on Best Practices for pig welfare

**DOI:** 10.3389/fvets.2026.1905492

**Published:** 2026-09-23

**Authors:** Maite Jachens, Swetlana Herbrandt, Nicole Kemper, Michaela Fels

**Affiliations:** 1 Institute for Animal Hygiene, Animal Welfare and Farm Animal Behavior, University of Veterinary Medicine Hannover, Foundation, Hannover, Germany; 2 Statistical Consulting and Analysis, Center for Higher Education, TU Dortmund University, Dortmund, Germany

**Keywords:** animal welfare, Best Practices, pigs, stakeholder comparison, surveys

## Abstract

**Background:**

Animal welfare presents an important topic in pig farming, with numerous resources offering recommendations on welfare improvements. These resources are often based on scientific knowledge, while practical experiences from farmers are rarely considered. This study aimed to bridge this gap in research by comparing scientific experts’ and farmers’ perceptions of Best Practices to improve on-farm welfare of fattening pigs. Candidate Best Practices to prevent and reduce tail and ear lesions, skin lesions and bursitis were identified in a literature review and integrated into two online surveys aimed at scientific experts and farmers. Participants could evaluate these measures on a scale from 0% (unsuitable) to 100% (very suitable). Answers were compared between both groups using a mixed linear regression model.

**Results:**

Overall, 47 scientific experts and 80 pig farmers participated in the surveys. Nearly all proposed Best Practices received a higher rating from scientific experts than from farmers. Nevertheless, the highest and lowest rated measures were similar for both groups and numerous measures did not show a significant difference between farmers and scientific experts (*p* > 0.05). Top-rated candidate Best Practices included “Good barn climate/air quality” (median experts: 84.0% vs. median farmers: 72.0%, *p* = 1.000) as well as “Good health status” (81.5% vs. 75.0%; *p* = 1.000) for tail and ear lesions and “As little regrouping/mixing of animals on farm as possible” (88.0% vs. 71.0%, *p* = 0.006) for skin lesions. Labor and cost-intensive candidate Best Practices such as “Lowering stocking density” (71.5% vs. 40.0%, *p* < 0.001) and “Enough feeding space per pig” (80.0% vs. 46.0%; *p* < 0.001) were scored significantly lower by farmers than by scientific experts. For bursitis, all proposed measures concerning floor condition showed a significant difference between the two groups (*p* < 0.05), with farmers again scoring all candidate Best Practices lower than scientific experts.

**Conclusion:**

While scientific experts favored most measures, farmers gave lower scores, potentially due to their experience with practical constraints such as feasibility, workload and costs. The ranking of candidate Best Practices, however, was similar in both groups, indicating a certain consensus. To promote the implementation of animal welfare measures on farms, it therefore seems important to consider farmers’ opinions and concerns and provide them with appropriate support.

## 1 Introduction

In recent years, animal welfare has become an important topic for both people working with animals as well as for consumers. In a survey from 2023, 91% of European citizens rated the animal welfare of farm animals as important, with 52% even deeming it “very important” (1). Meanwhile meat consumption and production in the European Union (EU) is still on a high level; 20.6 million tons of pig meat were produced in the EU in 2023 (2). Worldwide, pork and other pig meat products are the second most consumed meats, only surpassed by poultry (3).

However, pig farming in particular presents many challenges for all stakeholders involved, especially concerning animal welfare. Since pigs are a species with complex cognitive abilities (4) and intricate needs, housing them adequately is a demanding task and requires good knowledge of their physiology and behavior. Several animal welfare issues such as tail biting (5–7), skin lesions (8, 9) or bursitis (10) underline the need for welfare-oriented animal husbandry and emphasize the importance of improvements in pig farming. At the same time, animal welfare has increasingly become a political issue, and initiatives such as the EU ban on farrowing crates for sows (11) have forced stakeholders to find animal welfare appropriate alternatives to previously common practices.

Such alternatives or recommendations to improve animal welfare are often referred to as “Best Practices.” This term describes approaches that are recognized to be particularly effective in a specific field (12). With regard to pig welfare, “Best Practices” refer to strategies that ensure the best possible conditions for pigs in terms of their welfare, health and behavioral needs. Resources such as the Five Domains Model (13) and the Welfare Quality® assessment protocols (14) offer guidance on important areas for welfare improvements, but there are also numerous other scientific publications that provide “Best Practices” for animal welfare. Most of them have been developed and published by researchers and governments and reflect the current state of scientific knowledge [e.g., (6, 15, 16)]. While studies on farmers’ perceptions of animal welfare exist [e.g., (17–19)], a direct comparison between scientific experts’ recommendations and farmers’ views is rarely made.

Rust et al. (20) further found that farmers felt a lack of empathy from scientists and governments and therefore put more trust in information they received from other farmers or agricultural organizations. Including both farmers’ as well as scientists’ views in Best Practices regarding animal welfare could enhance collaboration between the two groups and advance solutions to animal welfare problems by combining knowledge (21). Research could also benefit from practical views to help in the implementation of scientific recommendations and align theory and practice (22). Furthermore, animal welfare policies might have a better chance of acceptance by farmers when practical on-farm challenges are taken into consideration.

The present study aimed to provide new insights into the opinions of scientific experts and pig farmers on candidate Best Practices to reduce tail and ear lesions, skin lesions and bursitis in pigs, which are considered important animal welfare indicators. Based on a literature review, various candidate Best Practices were identified and subsequently incorporated into an online survey. Participants were able to evaluate the proposed Best Practices regarding their suitability.

All answers were analyzed and compared to identify alignments as well as divergences between pig farmers and animal welfare scientists.

## 2 Materials and methods

### 2.1 Literature review

The study commenced with a literature review, using the databases PubMed^1^ and Web of Science^2^ from September to December 2023. The animal welfare indicators “tail lesions,” “ear lesions,” “skin lesions” and “bursitis” were used as search terms in combination with the animal species “pig.” Additionally, for tail and ear lesions, the search terms “tail biting” as well as “ear biting” were applied to include other relevant publications. Only relevant scientific literature written in English and published between 1975 and 2023 was considered, including peer-reviewed scientific articles and other relevant academic and institutional publications, such as EFSA scientific opinion papers. In addition to the database searches, relevant sources identified through the reference lists of publications and considered relevant to the topic were included.

The titles of the identified publications were initially screened. Publications concerning species other than pigs or topics unrelated to the effects of measures on the aforementioned animal welfare indicators were excluded. This included publications addressing the prevalences, pathology and physiology of tail and ear lesions, skin lesions and bursitis without investigating measures to reduce their occurrence. Furthermore, as part of this screening process, duplicates were removed. The abstracts of the remaining publications were then screened. Only scientific references that contained measures that were shown to be effective in reducing the occurrence of the aforementioned animal welfare issues on farm or at the slaughterhouse were considered for this study.

The scientific publications identified during the literature research were collected in a Microsoft Excel sheet (V.16.0.5483.1000, Microsoft Cooperation, Redmond, WA, USA). The citations as well as the specific measures identified in the publications were listed for each animal welfare indicator. The identified measures were subsequently grouped into thematic categories, such as feeding management, enrichment and handling. A validation score from zero to three according to the number of publications recommending a specific measure was introduced (Table 1).

**Table 1:** Validation score for measures identified in the literature review.

Validation score	Number of scientific publications recommending this measure
0	1
1	2 to <5
2	5 to <10
3	10 or more

### 2.2 Survey design

The program LimeSurvey (V6.6.6 +2 41002 LimeSurvey GmbH, Hamburg, Germany) was used to create two surveys. One was aimed at pig farmers, while the other targeted animal welfare scientists. First, a welcome text as well as a privacy policy, informing participants about the use of their data for research purposes and the anonymous handling of the data, were presented. The survey for animal welfare experts consisted of two main parts: questions about the participant’s background (e.g., field of experience, work country) and an evaluation of suggested Best Practices to reduce the occurrence of tail/ear lesions, skin lesions and bursitis in fattening pigs. The suggested Best Practices were measures that received a high validation score (2 and 3) in the literature review and were therefore considered important.

The survey for pig farmers consisted of three main parts: questions about the participant’s background (e.g., education, work country, employment status), an evaluation of how often participants had encountered specific animal welfare issues in pigs on their farms and an evaluation of candidate Best Practices to reduce tail/ear lesions, skin lesions and bursitis. A Likert scale (never/rarely/sometimes/often) was used to indicate the frequency of encountering the welfare issues tail/ear biting, skin lesions and bursitis on farm. In both surveys, the proposed measures (candidate Best Practices) could be evaluated on a scale from 0 to 100% with the use of a slider. A score of 0% equaled a measure that is unsuitable for animal welfare improvements, while a score of 100% was equivalent to a very suitable measure. The complete survey templates are included in the Supplementary material. It was not mandatory to evaluate every proposed Best Practice to ensure that participants only evaluated candidate Best Practices with which they had experience. Consequently, the number of answers for each question might deviate from the total number of participants. Prior to distribution, a preliminary version of both surveys was tested with 15 persons that were not part of either respondent group. The results from these tests were not included in the final analysis.

### 2.3 Survey distribution

The questionnaire for animal welfare experts was sent out to all project partners of the EU Horizon project “aWISH” (Animal Welfare Indicators at the Slaughterhouse), to the project’s expert panel as well as to 128 animal scientists from 21 countries all over the world. The expert panel contained the categories “Livestock sector,” “Industry & Retail”, “Government & Non-profit organizations (NGOs)” and “Research.” For each category, stakeholders could register via the project website, providing their position, expertise and references. Suitable candidates were then selected by the designated expert panel lead. The external scientific experts that were not part of the aWISH project nor the expert panel were chosen regarding three criteria: a current employment in research and/or academia, at least post-doc status in their current role and at least one publication on pig welfare in the last 5 years prior to the survey distribution. The partners of the aWISH project and the expert panel were not specifically chosen based on the same criteria; however, only those with in-depth scientific knowledge of pig welfare were encouraged to participate in the survey. Therefore, they were additionally asked in the survey whether they were scientifically experienced with pigs. Only participants answering “Yes” to this question could continue with the evaluation of candidate Best Practices. From 26 February 2024 to 15 March 2024, the survey was available to project partners and the expert panel. It was then sent out to the external experts who were able to participate between 19 March and 5 April 2024.

The survey with pig farmers was available from 4 June 2024 to 31 October 2024. First, a sample of individual German pig farmers were contacted via e-mail on their business websites and via the German chambers of agriculture. Additionally, several pig organizations in Germany, Austria and Switzerland were asked to distribute the survey to their associated farmers. A hyperlink and a QR code of the survey were also published on the website of the German agricultural magazine “top agrar.”

### 2.4 Statistical analysis

All data received in the surveys were downloaded from LimeSurvey (V6.6.6 +2 41002 LimeSurvey GmbH, Hamburg, Germany) and exported to Microsoft Excel (V.16.0.5483.1000, Microsoft Cooperation, Redmond, WA, USA). The software R 4.3.1 was used for the statistical analysis (23).

A descriptive analysis was performed for both surveys, taking into account all collected variables. For multiple-choice questions (e.g., background) as well as for questions containing a Likert scale, frequency distributions (percentages) were calculated. For the evaluation of measures based on a continuous scale of agreement (0–100%), means and medians as well as standard deviations were assessed. Subsequently, a mixed linear regression model (24) was applied to the evaluation of measures to display their perceived effectiveness in relation to the type of measure and the respondent group. The level of significance was set at *p* < 0.05. In the model, type of measure, group (expert or farmer) and their interactions represented fixed effects, while individual respondents were considered as a random effect. Model adjustment was done using the R package lmerTest (25). The R package emmeans (26) was applied to perform pairwise comparisons between experts and farmers, with *p*-values adjusted for multiple testing using the Bonferroni method.

For statistical purposes, some changes were made to the original data in two questions: Due to small numbers of scientific experts that chose “Technology provider” or “Industry” as their current position, those backgrounds were added to the option “Other.” For the same reason, the options “Hauptschulabschluss” and “Realschulabschluss” (both school-leaving qualifications in Germany) as well as “high school diploma” in the survey with farmers were combined so that the group “school diploma” included all participants that selected one of these options.

## 3 Results

### 3.1 Literature review

A total of 109 scientific publications were identified during the literature review: 62 for the animal welfare indicator tail and ear lesions, 30 for skin lesions and 17 for bursitis.

For tail and ear lesions, many measures were reported to influence the occurrence of this animal welfare indicator. Environmental enrichment, provision of straw, lowering stocking density, good health status as well as good barn climate were reported frequently and thus received a validation score of 3. Furthermore, several measures relating to the topic of feeding were often recommended and received a validation score of 2 (Table 2).

**Table 2:** Best Practices affecting different animal welfare indicators identified in the literature and their validation score based on the frequency of their recommendation.

Animal welfare indicator	Identified Best Practices	Validation score	References
Tail and ear lesions	Environmental enrichment and changing pen design	3	(7, 15, 27–54)
	Provision of straw	3	(7, 15, 27, 30, 37–40, 45–47, 49, 55–58)
	Lowering stocking density	3	(7, 15, 19, 27, 30, 31, 33, 34, 44, 48, 51–53, 59–61)
	Good barn climate	3	(7, 15, 19, 39, 41, 44, 45, 47–49, 59–63)
	Good health status	3	(7, 15, 19, 27, 39, 41, 43–45, 47, 48, 64)
	Changing group size	2	(15, 31, 44, 48, 65–67)
	Little mixing/regrouping of pigs	2	(6, 43, 48, 68–72)
	Good feed quality	2	(15, 19, 44, 47, 48, 51, 61, 73, 74)
	Enough feeding space per pig	2	(7, 27, 39, 43, 45, 75)
	Good feeding management	2	(39, 42–44, 47, 49, 60)
	Genetic aspects	2	(38, 48, 76–78)
	Bedding	2	(15, 39, 52, 55–57)
	Automated monitoring systems	1	(15, 79, 80)
	Floor design (more solid flooring)	1	(15, 31, 43)
	Good water access	1	(39, 41, 45)
	Good hygiene	1	(64, 73)
Skin lesions	Little mixing/regrouping of pigs	3	(8, 71, 81–88)
	Environmental enrichment and changing pen design	2	(52, 84, 89–95)
	Lower stocking density	2	(8, 52, 93, 95–98)
	Changing group size	2	(8, 97–100)
	Gentle handling	2	(8, 98, 99, 101, 102)
	Feeding	1	(8, 82, 95, 103)
	Short lairage stays	1	(8, 9, 85, 104)
	Small groups in lairage	1	(8, 98, 99)
	Early life socialization	1	(83, 105)
	Transport	1	(85, 106)
	Loading	1	(8, 87)
Bursitis	Provision of bedding	3	(58, 107–117)
	Changing floor type:	3	(109, 110, 115, 118, 119)
	Soft/soil floors		
	Solid instead of slatted floors		(107, 109, 110, 120)
	Rubber floor mats		(113, 121, 122)
	When using slats: plastic instead of metal or concrete		(109, 113)
	Good floor condition	2	(107, 109, 110, 114, 118)
	Extensive husbandry	1	(112, 117)

Little mixing or regrouping of pigs was the only measure that received a validation score of 3 for the animal welfare indicator skin lesions. Other recommended measures reducing skin lesions were environmental enrichment, careful handling, lower stocking density and group size which were assigned the score 2 (Table 2).

Concerning the occurrence of bursitis, most identified scientific papers included measures revolving around the flooring in the pens. Three measures were determined to influence bursitis in pigs: bedding and changing floor type (both with a validation score of 3) as well as a good floor condition in general (score 2; Table 2).

### 3.2 Survey

Overall, 130 animal welfare experts as well as 160 pig farmers participated in the two surveys. Only fully completed questionnaires from suitable candidates were included. Questionnaires where the survey was stopped before the end-screen as well as those from unsuitable candidates (e.g., a non-farming veterinarian participating in the survey for farmers) were excluded, leaving a total of 127 completed questionnaires for analysis (47 animal welfare experts and 80 pig farmers). The number of participants for each answer may differ, since not all questions were answered by every participant. Participants were encouraged to evaluate only candidate Best Practices they were familiar with and to use the “reset” button for any they did not feel knowledgeable enough to assess.

#### 3.2.1 Participants’ background

##### 3.2.1.1 Scientific experts

Most scientific experts (63.8%) were working in research/academia, followed by consulting (14.9%), slaughterhouse (12.8%) and other fields such as NGOs or veterinary associations (8.5%). As shown in Figure 1, experts had the most experience in animal production/agriculture (median: 19 years) and animal behavior/animal welfare (median: 18 years). Experience in other fields, e.g., precision livestock farming, was indicated by 29.8% of participants.

**Figure 1:**
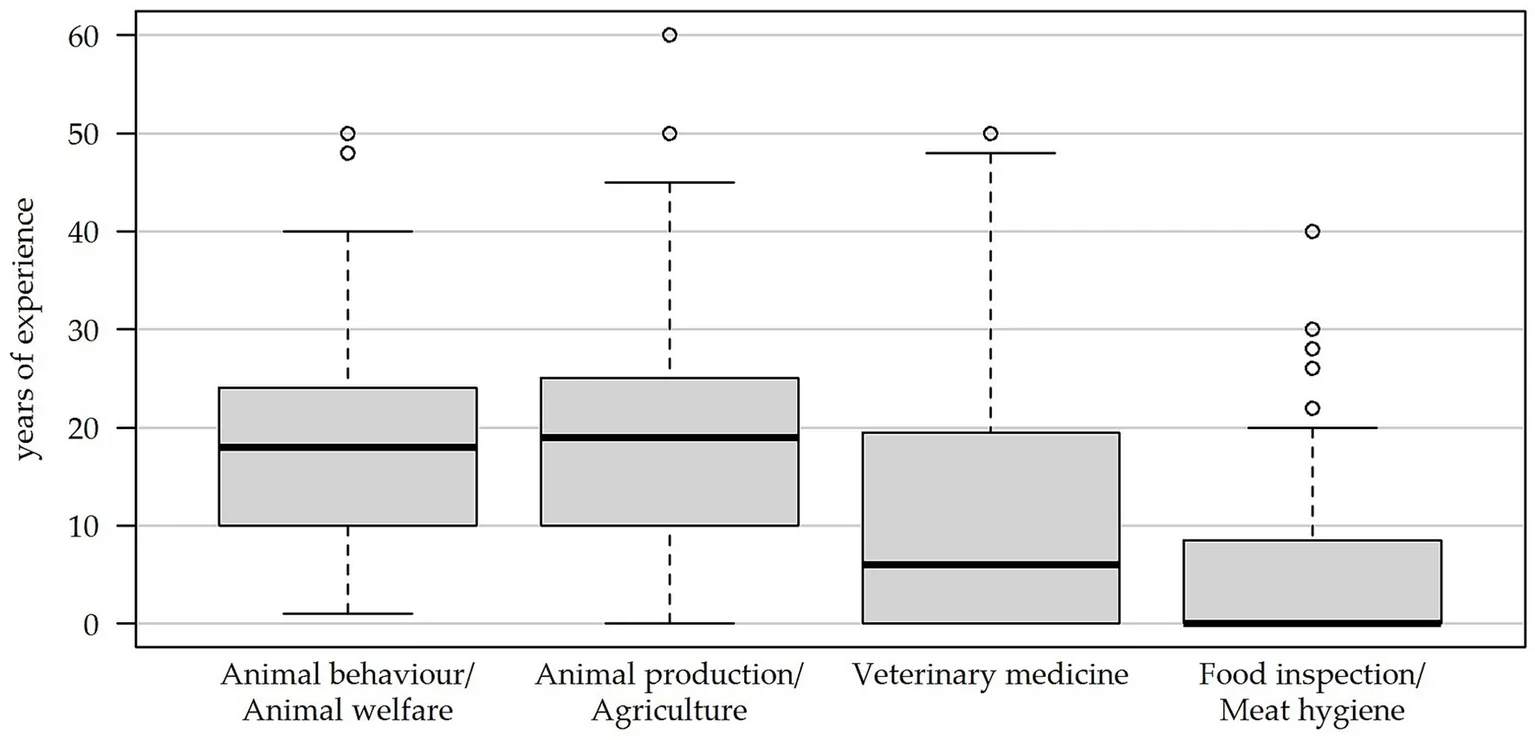
Years of experience of experts participating in the survey, *n* = 47 experts.

##### 3.2.1.2 Farmers

The majority of farmers that participated in the survey were self-employed (77.5%). A total of 8.8% of participating farmers were employed on a farm and 7.5% worked as agricultural service providers, amounting to 13 participants in total. 6.3% of participating farmers also indicated other backgrounds (e.g., agricultural students). Their years of experience ranged from 1 to 50 years, with a median experience of 27 years. From Figure 2 it is evident that self-employed farmers showed the greatest amount of experience in their field (median: 30 years) compared to employed farmers (median: 17 years) and agricultural service providers (median: 25 years). A total of 43.8% of participating farmers held a university degree (Bachelor’s, Master’s), followed by 30% with a German Meisterbrief (master craftsman), 10% with a school diploma, 8.8% with completed vocational training and 7.5% with a doctoral degree/PhD. A total of 60% of the participating farmers stated that they worked exclusively with pigs. Most farmers also working with other animals selected cattle (75%) or laying hens (40.6%) as additional species. Since the survey for farmers was mainly distributed in Germany, the vast majority of participants (90%) were German. All other participants selected Austria as their work country. Furthermore, the frequency of how often farmers encountered the proposed animal welfare indicators was evaluated (Table 3). The majority of participants had experienced tail and ear biting sometimes (47.5%) or even often (28.8%). Only 2.5% of participants had never experienced tail or ear biting on farm. Skin lesions were encountered rarely (38%) or sometimes (38%), but a considerable amount of participants (20.3%) also came across these lesions often. Only 3.8% had never experienced these lesions. As for bursitis, about half of the participating farmers (52.5%) stated that they had encountered this animal welfare issue rarely. However, a quarter (25%) also selected “sometimes.” At 11.3%, both “never” as well as “often” were chosen equally.

**Figure 2:**
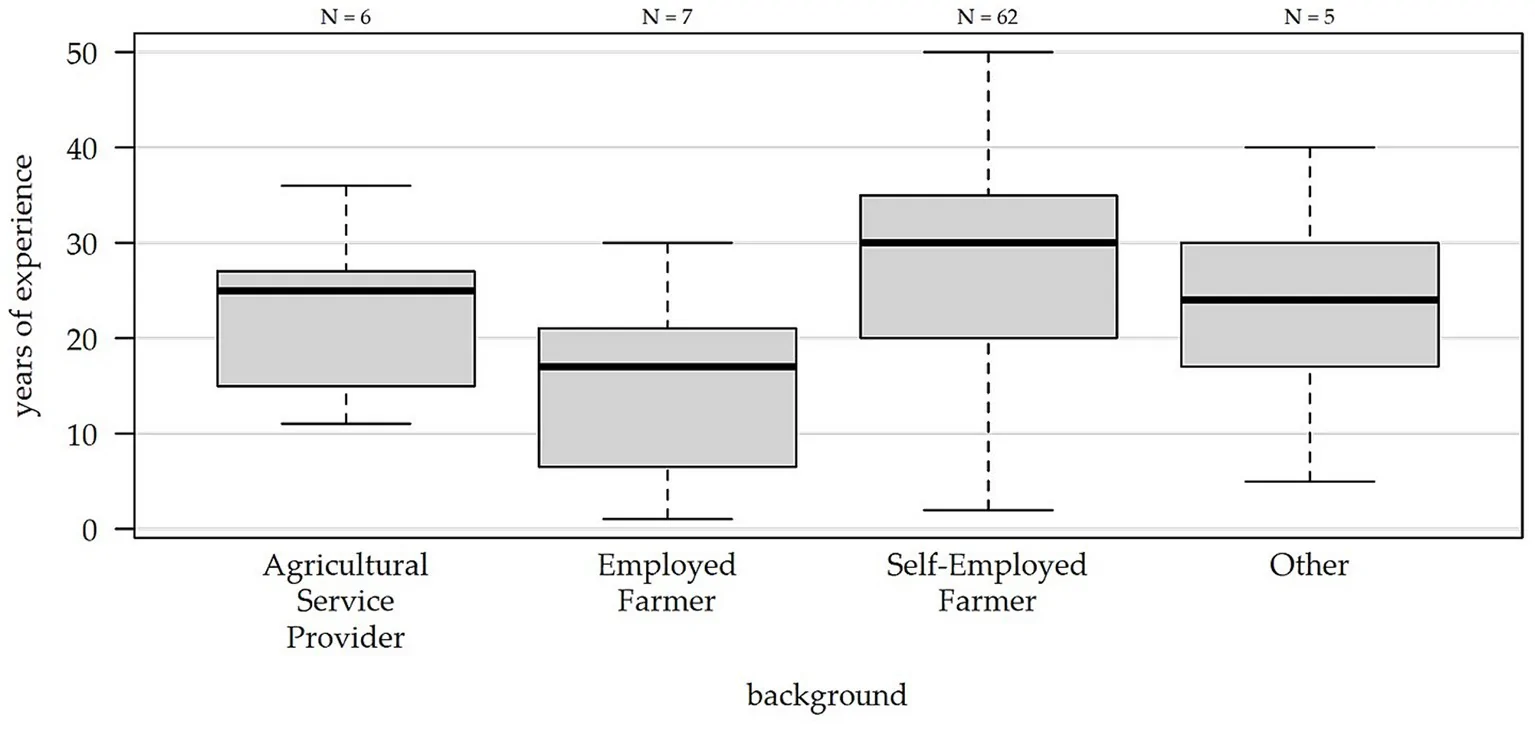
Years of experience depending on background of farmers participating in the survey, *n* = 80 farmers.

**Table 3:** Experience of farmers with animal welfare issues stated in the survey, *n* = 80 farmers.

Animal welfare issues	Animal welfare issues experienced in pigs			
	Never	Rarely	Sometimes	Often
Tail/ear biting	2 (2.5%)	17 (21.3%)	38 (47.5%)	23 (28.8%)
Skin lesions	3 (3.8%)	30 (38.0%)	30 (38.0%)	16 (20.3%)
Bursitis	9 (11.3%)	42 (52.5%)	20 (25.0%)	9 (11.3%)

#### 3.2.2 Tail/ear lesions

Experts rated most proposed measures for the animal welfare indicator “tail and ear lesions” as suitable. Only four out of the 21 candidate Best Practices received median ratings for the level of agreement below 50% (on a scale from 0 to 100%): “Non-organic manipulable material on farm (metal chains, chewable plastic material, etc.)” (40.0%), “Very small group size on farm (similar to a litter size: 10 – 16 pigs)” (40.0%), “Small group size on farm (<40 pigs per group)” (40.0%) and “Bigger group size on farm (>40 pigs per group)” (17.5%). However, as shown in Figure 3, individual responses showed a large variance, since most measures had a minimum agreement of 0% and a maximum of 100%. All proposed measures and their answer codes to prevent tail/ear lesions are listed in Table 4.

**Figure 3:**
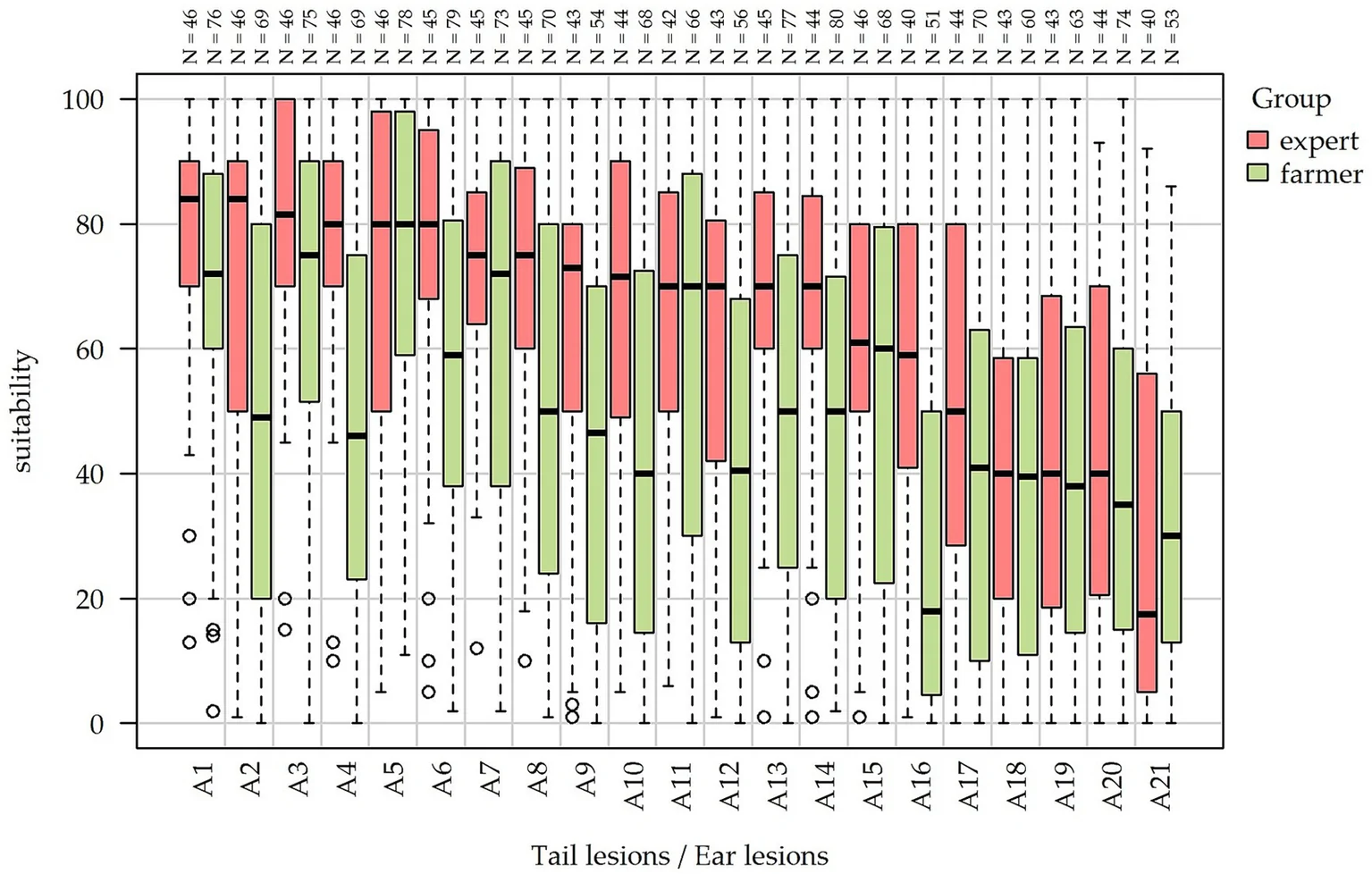
Level of agreement (0–100%) for measures to prevent tail/ear lesions (experts vs. farmers; A1–A21: see Table 4).

**Table 4:** Explanation of answer codes (Best Practices) for tail and ear lesions used in Figure 3 and visualization of the top three Best Practices for experts and farmers.

Tail lesions/ear lesions		Top BPs experts	Top BPs farmers
A1	Good barn climate/air quality (e.g., little ammonia, optimal air flow, etc.)	1	3
A2	Provision of straw as bedding on farm	2	
A3	Good health status	3	2
A4	Enough feeding space per pig (preferably 1:1)		
A5	Good feed quality (e.g. no mycotoxins, balanced)		1
A6	Regular refilling of manipulable or bedding material on farm (so it does not run out)		
A7	Good feeding management (e.g. ad libitum, short feeding intervals)		
A8	Structuring of the pen (lying, feeding, dunging area)		
A9	Keeping litter mates together from birth to the fattening/finishing period (no mixing)		
A10	Lowering stocking density below EU legislation/more space allowance per pig		
A11	Genetic aspects (genetics that are preferred in countries without tail docking)		
A12	Pens with outdoor area		
A13	Provision of straw or hay as enrichment (in racks, feeders, etc.)		
A14	Organic manipulable material on farm except straw (wood, sisal ropes, pressed molasses, etc.)		
A15	As little regrouping/mixing of animals on farm as possible		
A16	No storage of manure in the stable (under the slatted floor)		
A17	Stocking density on farm according to EU legislation		
A18	Small group size on farm (<40 pigs per group)		
A19	Very small group size on farm (similar to litter size: 10–16 pigs)		
A20	Non-organic manipulable material on farm (metal chains, chewable plastic material, etc.)		
A21	Bigger group size on farm (>40 pigs per group)		

A similar variance in individual responses was also detected in the survey with farmers. In contrast to the experts, farmers rated more than half (11 out of 21) of the proposed Best Practices as less suitable, with a median rating of the level of agreement below 50%. Only five measures scored a median rating of agreement above 70%: “Good health status” (75.0%), “Good barn climate/air quality (e.g. little ammonia, optimal air flow, etc.)” (72.0%), “Good feed quality (e.g. no mycotoxins, balanced)” (80.0%), “Good feeding management (e.g. ad libitum, short feeding intervals)” (72.0%) and “Genetic aspects (genetics that are preferred in countries without tail docking)” (70.0%). Figure 3 shows the results of the responses for each measure.

Overall, scientific experts gave a higher level of agreement to the measures than farmers, as indicated in Figure 3. Pairwise comparisons between farmers and scientific experts showed a statistically significant difference for eight out of the 21 proposed answers (*p* < 0.05, Table 5). Large differences were seen for the measures “Organic manipulable material on farm except straw” (median experts: 70.0% vs. median farmers: 50.0%), “Lowering stocking density below EU legislation” (71.5% vs. 40.0%) and “Enough feeding space per pig” (80.0% vs. 46.0%; all *p* < 0.001).

**Table 5:** Results of pairwise comparisons between experts and farmers for the level of agreement regarding measures to prevent tail and ear lesions.

Tail lesions/ear lesions	Estimate	SE	*df*	*t* ratio	*p* value	2.5%	97.5%
A1—barn climate	8.973	5.295	733.136	1.695	1.000	−7.169	25.116
A2—straw as bedding	20.056	5.364	763.267	3.739	0.004	3.704	36.408
A3—health status	9.708	5.304	737.077	1.830	1.000	−6.461	25.878
A4—enough feeding space	29.533	5.364	763.295	5.505	<0.001	13.181	45.885
A5—feed quality	−0.612	5.277	725.486	−0.116	1.000	−16.700	15.477
A6—refilling of manipulable material	16.908	5.293	732.663	3.194	0.031	0.771	33.046
A7—feeding management	9.645	5.348	756.235	1.803	1.000	−6.658	25.948
A8—structuring of the pen	19.976	5.379	769.571	3.714	0.005	3.580	36.372
A9—keeping littermates together	17.128	5.642	887.169	3.036	0.052	−0.063	34.318
A10—lowering stocking density	23.025	5.426	790.341	4.243	<0.001	6.487	39.563
A11—genetic aspects	5.306	5.503	824.570	0.964	1.000	−11.464	22.076
A12—outdoor area	16.938	5.610	872.530	3.019	0.055	−0.155	34.031
A13—straw or hay as enrichment	17.005	5.311	740.110	3.202	0.030	0.815	33.195
A14—organic manipulable material	22.500	5.309	741.672	4.238	<0.001	6.316	38.685
A15—little regrouping/mixing	9.038	5.375	768.095	1.681	1.000	−7.348	25.423
A16—no storage of manure	23.398	5.778	949.261	4.049	0.001	5.795	41.000
A17—stocking density = EU	12.900	5.405	780.888	2.387	0.362	−3.574	29.373
A18—small group size	5.215	5.551	846.123	0.939	1.000	−11.701	22.132
A19—very small group size	4.547	5.512	828.426	0.825	1.000	−12.251	21.344
A20—non-organic manipulable material	4.101	5.364	763.378	0.765	1.000	−12.250	20.452
A21—bigger group size	−2.610	5.743	933.174	−0.454	1.000	−20.106	14.886

SE, standard error; *df*, degrees of freedom; 2.5 and 97.5%, lower and upper confidence interval limits.

For experts, the top three candidate Best Practices included “Provision of straw as bedding on farm” (median: 84.0%), “Good barn climate/air quality” (median: 84.0%) and “Good health status” (median: 81.5%). The proposed Best Practices “Good barn climate/air quality” (median: 72.0%) and “Good health status” (median: 75.0%) were also among the top three candidate Best Practices for farmers. However, “Good feed quality” (median: 80.0%) was the number one candidate Best Practice for this respondent group. In both groups, all proposed pig group sizes (very small, small and bigger groups) received scores below 50% that did not differ greatly. Certain proposed Best Practices such as “As little regrouping/mixing of animals on farm as possible” (median experts: 61.0% vs. median farmers: 60.0%) and “Non-organic manipulable material on farm” (40.0% vs. 35.0%) received similar ratings in both respondent groups.

#### 3.2.3 Skin lesions

Similar to the results for tail and ear lesions, scientific experts only rated three out of the 14 proposed measures with a median level of agreement below 50% (Figure 4). The three candidate Best Practices “As little regrouping/mixing of animals on farm as possible” (median: 88.0%), “Keeping littermates together from birth to fattening/finishing period (no mixing)” (median: 90.0%) and “Gentle animal handling on farm” (median: 80.5%) received a median score above 80%. All proposed measures to prevent tail/ear lesions and their answer codes are listed in Table 6.

**Figure 4:**
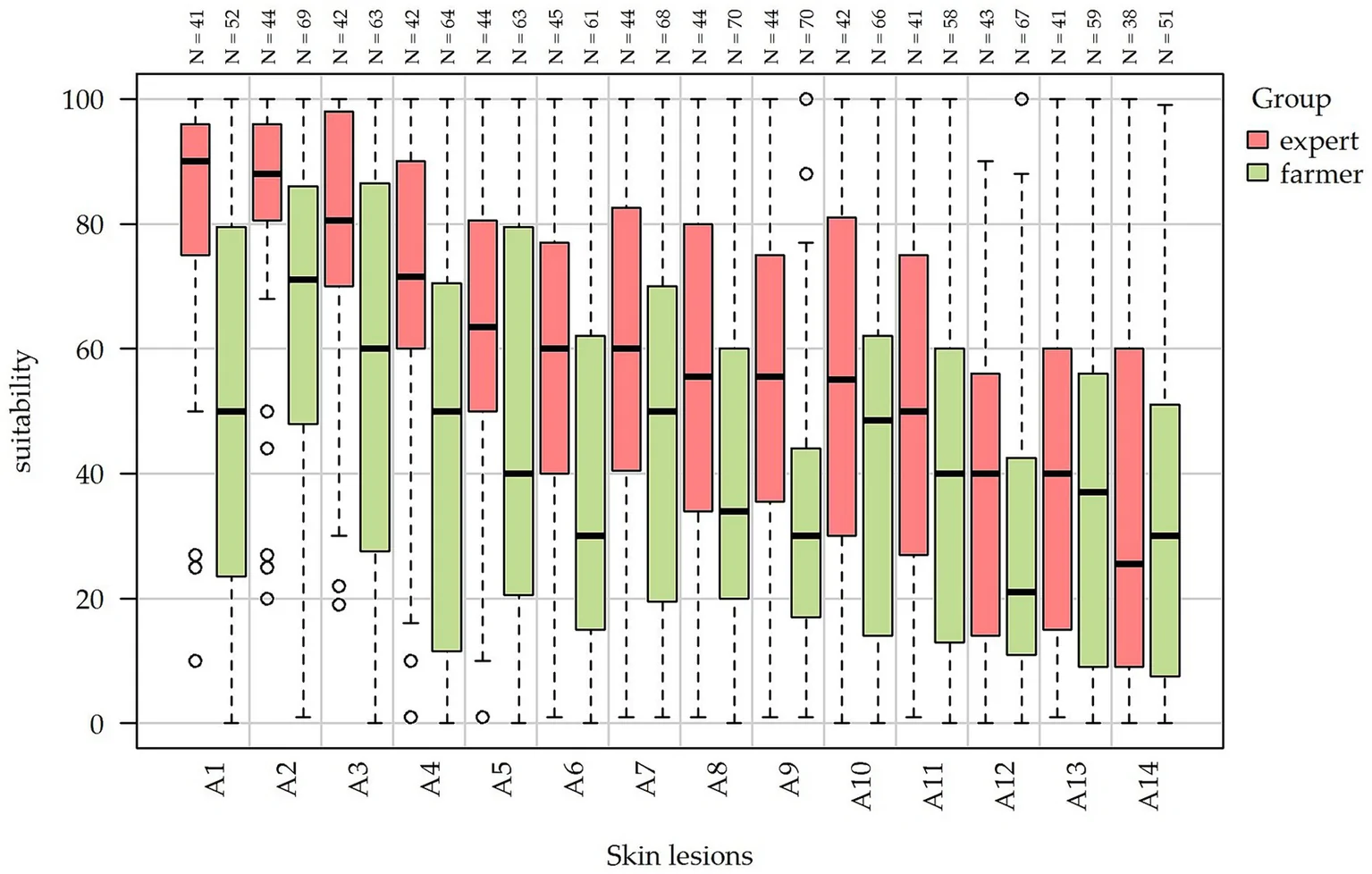
Level of agreement (0–100%) for measures to prevent skin lesions (experts vs. farmers; A1–A14: see Table 6).

**Table 6:** Explanation of answer codes for skin lesions used in Figure 4 and visualization of the top three Best Practices for experts and farmers.

Skin lesions		Top BPs experts	Top BPs farmers
A1	Keeping littermates together from birth to fattening/finishing period (no mixing)	1	3
A2	As little regrouping/mixing of animals on farm as possible	2	1
A3	Gentle animal handling on farm	3	2
A4	Lowering stocking density on farm below EU legislation/more space allowance per pig		
A5	Structuring of pens (lying, feeding, dunging area)		
A6	Provision of straw as bedding on farm		
A7	Regular refilling of manipulable or bedding material on farm (no running out)		
A8	Provision of straw or hay on farm as enrichment (in racks, feeders, etc.)		
A9	Organic manipulable material on farm except straw (wood, sisal ropes, pressed molasses, etc.)		
A10	Stocking density on farm according to EU legislation		
A11	Very small group size on farm (similar to litter size: 10–16 pigs)		
A12	Non-organic manipulable material on farm (metal chains, chewable plastic material, etc.)		
A13	Small group size on farm (< 40 pigs per group)		
A14	Bigger group size on farm (> 40 pigs per group)		

In the farmers’ survey, nine of the proposed Best Practices received a median level of agreement below 50%. Only two measures, “As little regrouping/mixing of animals on farm as possible” (median: 71.0%) and “Gentle animal handling on farm” (median: 60.0%), obtained a median percentage of agreement of 60% or more. Individual responses showed a large variance for this animal welfare indicator, too (Figure 4). Similar to tail/ear lesions, scientific experts overall ranked all measures higher than farmers, with the exception of “Bigger group size on farm” (median experts: 25.5% vs. median farmers: 30.0%). Table 7 gives an overview of the results of all pairwise comparisons between experts and farmers.

**Table 7:** Pairwise comparison experts vs. farmers for skin lesions.

Skin lesions	Estimate	SE	*df*	*t* ratio	*p* value	2.5%	97.5%
A1—keeping littermates together	29.575	5.784	601.065	5.114	<0.001	12.656	46.494
A2—little regrouping/mixing	19.557	5.487	514.507	3.564	0.006	3.494	35.620
A3—gentle animal handling	19.903	5.602	547.917	3.553	0.006	3.510	36.296
A4—lowering stocking density	22.803	5.593	544.786	4.077	<0.001	6.435	39.171
A5—structuring of pens	12.430	5.555	533.486	2.238	0.359	−3.829	28.690
A6—straw as bedding	17.642	5.555	533.596	3.176	0.022	1.384	33.900
A7—refilling of manipulable material	10.407	5.499	517.384	1.893	0.825	−5.688	26.502
A8—straw or hay as enrichment	16.105	5.479	511.670	2.940	0.048	0.067	32.143
A9—organic manipulable material	19.660	5.476	512.165	3.590	0.005	3.629	35.691
A10—stocking density = EU	13.841	5.570	538.184	2.485	0.186	−2.460	30.142
A11—very small group size	10.189	5.695	574.497	1.789	1.000	−6.474	26.853
A12—non-organic manipulable material	6.080	5.535	527.698	1.098	1.000	−10.121	22.281
A13—small group size	8.056	5.682	570.592	1.418	1.000	−8.568	24.680
A14—bigger group size	−0.099	5.887	631.572	−0.017	1.000	−17.317	17.118

SE, standard error; *df*, degrees of freedom; 2.5 and 97.5%, lower and upper confidence interval limits.

The top two candidate Best Practices for both respondent groups were the same, although the level of agreement varied considerably between scientific experts and farmers: “As little regrouping/mixing of animals on farm as possible” (median experts: 88.0% vs. median farmers: 71.0%, *p* = 0.006) and “Gentle animal handling on farm” (80.5% vs. 60.0%, *p* = 0.006). Again, all proposed group sizes did not receive median scores above 50% in either of the respondent groups.

#### 3.2.4 Bursitis

As shown in Figure 5, for bursitis, the scientific experts’ agreement to all six proposed Best Practices was above 50%. “Non-slatted concrete floor” received the lowest median level of agreement (59.0%), while “Bedding (e.g. straw)” obtained the highest agreement (87.0%). In Table 8, the proposed Best Practices and their answer codes are listed.

**Figure 5:**
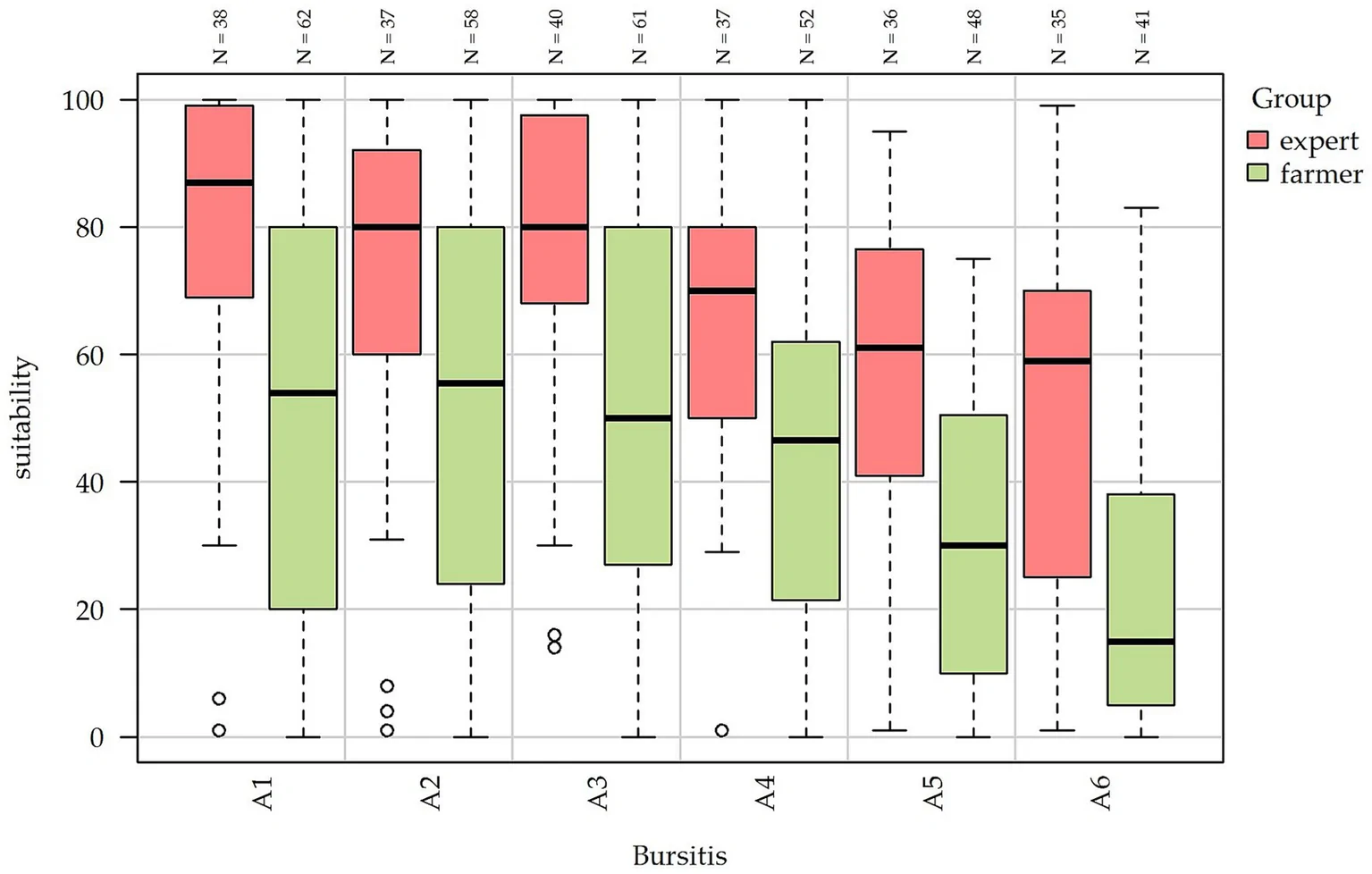
Level of agreement (0–100%) for measures to prevent bursitis (experts vs. farmers; A1–A6: see Table 8).

**Table 8:** Explanation of answer codes for bursitis used in Figure 5 and visualization of the top three Best Practices for experts and farmers.

Bursitis		Top BPs experts	Top BPs farmers
A1	Bedding (e.g. straw)	1	2
A2	Soft or soil floors, no slats	2	1
A3	Good floor condition (e.g. no breakage, non-slippery)	3	3
A4	Rubber floor mats		
A5	When using slats: plastic rather than concrete or metal ones		
A6	Non-slatted concrete floor		

Farmers rated half of the measures below 50% and half of them above 50%. However, the highest rated measure “Soft or soil floors, no slats” only received a median level of agreement of 55.5%. The candidate Best Practice “Non-slatted concrete floor” received the lowest median agreement of all measures in the survey (15.0%; Figure 5).

All proposed Best Practices showed a statistically significant difference in the agreement between farmers and experts (Table 9). Experts again gave overall higher agreement to all measures than farmers. The top three candidate Best Practices for both respondent groups were identical: “Bedding (e.g., straw)” (median experts: 87.0% vs. median farmers: 54.0%, *p* < 0.001), “Soft or soil floors, no slats” (80.0% vs. 55.5%, *p* = 0.008) and “Good floor condition” (80.0% vs. 50.0%, *p* < 0.001). Furthermore, both groups showed the same tendency in the assessment of candidate Best Practices (from high to lower ranking measures; Figure 5).

**Table 9:** Pairwise comparison experts vs. farmers for bursitis.

Bursitis	Estimate	SE	*df*	*t* ratio	*p* value	2.5%	97.5%
A1 bedding	25.041	5.797	375.015	4.320	<0.001	9.665	40.416
A2—soft or soil floors	18.998	5.904	383.937	3.218	0.008	3.342	34.654
A3—floor condition	23.487	5.733	370.375	4.097	<0.001	8.280	38.694
A4—rubber floor mats	25.511	6.012	394.571	4.244	<0.001	9.571	41.451
A5—plastic slats	27.483	6.133	406.942	4.481	<0.001	11.223	43.742
A6—non-slatted floor	25.423	6.346	428.156	4.006	<0.001	8.601	42.245

SE, standard error; *df*, degrees of freedom; 2.5 and 97.5%, lower and upper confidence interval limits.

## 4 Discussion

Animal welfare issues such as tail lesions, skin lesions or bursitis in pigs, including Best Practices to prevent or mitigate these problems, have been explored in a number of studies [e.g., (60, 86, 109)]. Thus, recommended measures to prevent lesions or bursitis in pigs are based on scientists’ findings rather than on farmers’ experience. While studies on farmers’ views regarding animal welfare also exist (18, 59, 75), a direct comparison between the recommendations and experiences of farmers and scientific experts is rarely made. This study fills that gap, as scientists and farmers were surveyed in a standardized manner using a uniform questionnaire regarding the effectiveness of the same measures to improve pig welfare.

### 4.1 The study sample

The survey was completed by partners of the aWISH project and members of the project’s expert panel, as well as external researchers from various countries who were not affiliated with the project. To ensure that only individuals with expertise in pig welfare participated, strict criteria were applied to the selection of external scientists. They were required to hold a current academic position, be at least at postdoctoral level, and have published works relevant to the topic. The partners of the aWISH project and the expert panel already brought extensive scientific experience; therefore, they were not explicitly asked to meet the same selection criteria. However, they had to confirm in the questionnaire that they possessed sufficient scientific expertise regarding pigs. Furthermore, the survey was circulated only among the selected groups of people, since it was not made public. It was therefore ensured as much as possible that only individuals with strong expertise participated.

Farmers were contacted through various channels in order to reach as many people with experience in pig farming as possible. Aside from expertise in pig farming, no other restrictions were placed on recruitment, allowing farmers of different ages, from different regions, and with farms of varying sizes to participate. However, since participation in the survey was voluntary, the sample size may not depict the actual farming population of the DACH region (Germany, Austria, Switzerland). The majority of farmers participating in the survey were self-employed, which represents the most common employment status in agriculture both in the EU (123) as well as worldwide (124). Both farmers as well as scientific experts possessed plenty of experience in their respective fields. Since 33.2% of farmers in the EU were aged 65 years or over in 2024 (123), this might explain the great amount of experience farmers indicated in the survey.

Farmers were further asked to indicate how often they encountered different animal welfare issues on their farms. Tail and ear lesions were most frequently experienced; more than 70% of farmers encountered these lesions sometimes or even often, which is in line with an estimation of the European Food Safety Authority (6) stating that tail lesions occur in 30–70% of all farms in the EU. Skin lesions as well as bursitis were experienced less often, with most participants selecting “rarely” or “sometimes.” Since the appearance of skin lesions coheres with mixing of pigs into new groups (8), transport or overnight lairage stays (9), they often occur for a limited time or when the animals are no longer on farm and may therefore be overlooked or cannot even be seen by the farmer. Skin lesions may also not be perceived as relevant for welfare if they are not severe injuries and only superficial scratches are visible. In several studies, bursitis has been found to be common in pigs (10, 107, 109). However, since it is so common, the farmers in this study might not perceive these lesions as pathological.

### 4.2 Tail/ear and skin lesions

The literature review for tail and ear lesions provided several measures to improve animal welfare and reduce tail and ear biting. Since tail biting has been proven to have multifactorial causes (7, 125), interventions and their effectiveness can differ depending on the cause of the outbreak and individual factors like the farm or animals themselves. Therefore, farmers’ experiences may vary widely, which could have influenced their confidence in specific measures to prevent tail lesions. This is also likely to be reflected in lower levels of agreement with recommended practices compared to scientific experts. Nevertheless, both groups showed alignments in their views of most proposed measures: “Good health status” as well as “Good barn climate/air quality” were among the top three candidate Best Practices in both surveys. Experts further highlighted the importance of enrichment and straw (both as bedding and as manipulable material), while farmers only scored measures belonging to these categories at about 50%. Studies by Bracke et al. (59) and Benard et al. (126) found that farmers were skeptical toward the role of boredom in causing tail biting, thus deeming enrichment less important. Furthermore, research has shown that many farmers tend to equate animal welfare with animal health and growth (17, 126–128), without including behavioral needs in their definition. Feeding-related measures received high ratings throughout both surveys and were found in many scientific publications in the literature review. This is in line with studies by Tuyttens et al. (129) as well as Valros et al. (75) who also found farmers to perceive feeding as an important aspect of animal welfare. Significant differences between the two groups were detected for several candidate Best Practices, including “Organic manipulable material on farm except straw”, “Lowering stocking density below EU legislation” and “Enough feeding space per pig”. These were favored by scientific experts but rated lower by farmers. These measures require costly changes in the barn such as new feeding systems, which can lead to economic constraints for the farmers. Furthermore, the provision of organic manipulable material can increase the workload if material has to be exchanged regularly. These aspects may have influenced the farmers’ opinion on the measures, whereby animal welfare may also have been assessed against the background of practical feasibility.

The animal welfare indicator “skin lesions” revealed similar results regarding the comparison of scientific experts and farmers. The candidate Best Practice “As little regrouping/mixing of animals on farm as possible” received the highest score for both farmers as well as experts and was furthermore the measure most frequently reported in the literature. Numerous studies suggest little regrouping to avoid fighting between the animals [e.g., (8, 82, 86)], one of the main causes of skin lesions. Scientific experts further gave high scores to the measure “Keeping littermates together from birth to fattening/finishing period” (median: 90.0%), whereas this measure only received a median of 50.0% in the survey with farmers. Mixing different litters is a common practice in pig production. Therefore, keeping littermates together until the finishing period might be a hindrance for most fattening farms. Both farmers as well as experts only scored measures related to environmental enrichment at around 50%. Skin lesions are mainly caused by aggression between pigs (8, 130), and factors such as group composition might play a larger role in causing aggressive behavior than a lack of enrichment. In scientific literature, results are contradictory. Several authors [e.g., (102, 131–133)] also determined that enrichment did not have a significant effect on reducing skin lesions in pigs, whereas other studies found an effect [e.g., (52, 91, 94)].

For both tail and ear lesions as well as skin lesions, group size was found to have an effect according to the literature. Three different group sizes on farm were proposed as candidate Best Practices for the surveys: a very small group size (similar to litter size: 10–16 pigs), a small group size (< 40 pigs per group) and a bigger group size (> 40 pigs per group). Experts as well as farmers did not agree with any of them. This could be due to the unclear phrasing, since none of the group sizes indicated the space allowance the animals would have, the group composition or the mixing strategy. The minimum space requirements stipulated by EU legislation were assumed but not explicitly mentioned in the possible answers. Another explanation could be that group size simply does not have an effect on these two indicators under practical farming conditions. While some research indicates a correlation between group size and tail and ear lesions (65, 67) as well as skin lesions (98, 100), other studies found no evidence that group size has an effect on the incidence of lesions (134, 135). Increased space allowance, however, was proved to enhance pig welfare (33, 44, 52, 97), indicating that space allowance might be more important than group size. The bigger group size (> 40 pigs per group) received the lowest agreement of all three proposed group sizes in both surveys. In nature, wild boars live in smaller groups of one to 23 individuals (136). Therefore, smaller groups on farm might ensure that pigs can fulfil their natural behavioral needs.

### 4.3 Bursitis

The literature review regarding bursitis revealed candidate Best Practices related to flooring and bedding. All proposed measures resulted in a significantly different assessment between scientific experts and farmers. Since the farmers in this study rarely encountered bursitis, they might not be too familiar with practices to reduce this problem. Additionally, most proposed Best Practices for bursitis involved changes in the floor design which entails costly investments as well as an additional workload for the farmers. This might have led to a more negative evaluation of the measures in comparison to the scientific experts. The two most effective candidate Best Practices regarding bursitis in pigs according to the results of the surveys were soft floor alternatives: “Bedding (e.g. straw)” and “Soft or soil floors, no slats” received the highest scores from both farmers as well as scientific experts. Hard floors are known to increase the prevalence of bursitis (115), and many studies identified bedding as a measure to improve limb health in pigs (58, 107, 109). “Good floor condition” ranked third in both surveys. KilBride et al. (118) also stressed the importance of a dry, non-slip floor to avoid bursitis. “Non-slatted concrete floor” received the worst rating throughout the surveys in both groups despite several scientific publications identified in the literature review reporting it as effective in preventing bursitis [e.g. (109, 110, 120)]. Slats are widely used in pig farming with the intention to create good animal hygiene via spatial separation of excreta from animal areas (137). On a non-slatted concrete floor, excreta can accumulate and cause the floor to be more slippery, which can cause bursitis when pigs slip and the resulting injuries become infected (114, 118). As previously mentioned, hard surfaces are also associated with an increased risk of bursitis (115), which may explain the low scores received in the surveys. Overall, studies on bursitis and recommended practices to prevent this condition are scarce. Further research is needed to determine more risk factors and intervention strategies under practical farming conditions.

### 4.4 Differences between scientific experts and farmers

Scientific experts were overall more inclined toward the proposed measures, whereas farmers gave lower scores. Other studies have found a similar trend between farmers and scientists (41, 59, 61, 75). Benard et al. (126) and Kakanis et al. (61) observed a knowledge gap between the two groups that was difficult to bridge via dialog. However, this argument may be too simplistic, as it neglects the fact that farmers possess a different form of knowledge and skills, making them experts in their field (138). Furthermore, it is already known that farmers are actively searching for, and using, information on animal health and welfare, and choose the most useful and feasible methods for their animals (21). Balzani and Hanlon (127) identified a total of 11 internal and 15 external factors that influence farmers’ attitudes toward farm animal welfare, including economic viability and workload, but also empathy and personal attitude. Meanwhile, scientists should conduct their research independently of their personal attitudes and moral or social values; however, in their role as advising experts, they should take into account the societal impact of their recommendations (138). Nevertheless, these different approaches to animal welfare and to Best Practices may have led to the discrepancies between the two groups in this study. Regional as well as cultural differences might also play a role in this study. While the participating farmers mainly originated from Germany, scientific experts from 16 different countries both within and outside the EU participated in the survey. The perception of animal welfare differs between nations (139, 140) and practices regarding pig welfare vary depending on national law as well as cultural values (141). This might have led to some differences in ratings between both groups as well as to differences between the individual experts. Some measures that received high ratings from the expert group in this study only scored at around 50% in the farmer group. A reason for this might be the different working conditions of the two groups: scientific experts working in research are often able to undertake studies under optimal (lab) conditions, whereas farmers have to deal with practical restrictions, such as structural conditions of the barns or feasibility during the workflow. Furthermore, due to the commercial situation, farmers also take into account economic and labor outcomes of the proposed measure, as their income depends on the performance of their animals (142). Mahon et al. (21) emphasized the importance of facilitating knowledge exchange within and between different groups and integrating different perspectives to enhance animal health and welfare. Such different perspectives were highlighted in the present study through the surveys with scientific experts and farmers.

However, not only views between the groups differed, but there was also a discrepancy between the individual participants of each group. For most proposed measures, answers ranged from the minimum of zero to the maximum of 100 percent. Several circumstances might contribute to this phenomenon. As previously mentioned, regional and cultural differences influence the manner participants perceive animal welfare (139–141). Heleski et al. (143) further showed that gender, political views and religious beliefs have an effect on stakeholders’ attitudes toward animal welfare. Systematic errors during the processing of information in the brain also affect the decision-making of individuals, the so-called “cognitive bias” (144, 145). An example relevant to this study is confirmation bias, the effect of looking for evidence that supports one’s beliefs or prior experiences (144). Participants might have been inclined to overrate familiar practices while underrating unfamiliar ones. In surveys, some participants further tend to provide inaccurate responses. Response bias such as acquiescence bias, the tendency to agree with all statements in a survey (146), as well as nay-saying, the tendency to disagree throughout a survey (147), have to be considered as potential reasons for the range in answers in this study. Regarding the survey with farmers, beliefs and values in the perception of animal welfare and good animal husbandry practices may also have influenced responses (148). Farmers should not be considered a homogenous group as their perceptions differ depending on factors such as farm size and structure, adopted management practices and experience (149). Valros and Barber (41) further found that farmers’ perceptions of animal welfare improving measures depend on their experience with these practices. Farmers who used bedding tended to give higher scores for this measure than others who housed their pigs on floors without bedding. Due to different conditions on individual farms, Best Practices could also vary between farms. Another factor contributing to the large variance is the definition of effectiveness for the individual participant. While some participants might have strictly evaluated the impact of the Best Practices in improving the proposed animal welfare indicators, others might also consider economic constraints, workload and feasibility in their ratings.

Despite the identified differences, the comparison between scientific experts and farmers also showed alignments. Both groups had the same tendencies in their ratings. Furthermore, many measures did not show a significant difference between experts and farmers. The top three candidate Best Practices for each animal welfare indicator were the same or similar for both groups.

### 4.5 Limitations

This study is subject to several limitations. An important limitation of the study is that the farmer survey included only German-speaking individuals. Since cultural differences and national laws influence how animal welfare is perceived and implemented in pig farming (139, 141), a true comparison between scientific experts from different countries worldwide and mostly German farmers is difficult. Self-selection bias might have also occurred. E-mails sent around to participants as well as the welcome text of both surveys clearly referred to animal welfare as the main theme of the survey. Therefore, individuals who place a high priority on animal welfare might have been more inclined to participate, while persons who are not very worried about animal welfare issues might be underrepresented in the current study. It was certainly the intention of the study to obtain recommendations from farmers who are engaged in animal welfare activities, as they are expected to have valuable experiences in this area. Nevertheless, it must be noted that these recommendations may not be supported by a broader farmer population. Another limitation is the lack of definition for the word “suitability.” All participants were asked to evaluate the proposed Best Practices according to their suitability; however, we did not provide a clear definition of the word. While some participants might have merely evaluated the actual impact of a certain candidate Best Practice, others might have included other factors such as costs and workload into their evaluation. Similarly, some proposed Best Practices such as different group sizes and lowering stocking density were phrased rather broadly, and no specific numbers (e.g., percentages for the lowering of stocking density) were provided. Individual participants might have interpreted these candidate Best Practices differently, which reduces the comparability of individual evaluations for these measures. It must also be pointed out that an online survey cannot provide deeper insights into the motivations behind the responses of individual participants in the two groups. Thus, it can only be speculated that, for the farmers, the practical implementation and economic feasibility of the proposed measures may have played a role. In-depth personal interviews would be necessary to gain better insights into the motivations behind the participants’ views. It is therefore advisable to conduct qualitative, structured interviews in future studies.

## 5 Conclusion

Overall, scientific experts were more likely to agree with the proposed measures to reduce tail, ear and skin lesions as well as bursitis than farmers. Potential reasons included different working conditions of the two respondent groups as well as farmers taking into account practical realities relating to economic and labor outcomes. However, both groups showed the same tendencies in their ratings, indicating that farmers and experts share a similar view on the relevance of individual measures. The top three candidate Best Practices for tail and ear lesions according to scientific experts and farmers were “Good health status,” “Good barn climate/air quality” and “Good feed quality.” Measures influencing the animal welfare indicator skin lesions were mostly related to mixing and handling. Top three candidate Best Practices for this indicator in both groups included “As little regrouping/mixing of animals on farm as possible,” “Gentle animal handling” and “Keeping littermates together from birth until fattening/finishing period.” Nevertheless, the ratings for “Keeping littermates together” showed a large discrepancy between experts and farmers, with farmers not being convinced of this measure. Scientific publications on bursitis were scarce. Most measures found in the literature related to floor type and also made their way into the top three Best Practices: “Bedding (e.g., straw),” “Soft or soil floors, no slats” as well as “Good floor condition” received the best ratings in both groups. The results highlight the importance of including both scientists’ as well as farmers’ views when discussing measures to improve animal welfare. Promoting dialog not only between these two groups, but also among individual farmers can help to identify barriers as well as solutions for the implementation of welfare improving practices. Consultations by advisers may further help to find individual solutions for different farms. Information on costs, additional workload for Best Practices and possible effects on farm productivity should be considered when measures are recommended to farmers, or they are even implemented into law-making. Furthermore, incentives should be provided to encourage farmers to adopt Best Practices that are backed by scientific evidence. They might, for instance, benefit from governmental and financial support to implement suitable measures on farm.

## Data Availability

The raw data supporting the conclusions of this article will be made available by the authors, without undue reservation.

## Footnotes

1 www.pubmed.ncbi.nlm.nih.gov.

2 www.webofscience.com.
