# Supplementary material for: It’s all a matter of perspective—a comparison of scientific experts’ vs. farmers’ opinions on Best Practices for pig welfare
Source: Front Vet Sci. 2026 Sep 23;13:1905492. doi: 10.3389/fvets.2026.1905492 (PMC13643911; doi:10.3389/fvets.2026.1905492)
Supplement: Supplementary file 1 [file Table_1.DOCX]

Survey template for pig farmers

**Best Practices from the perspective of pig farmers**

**The EU project “Animal Welfare Indicators at the Slaughterhouse” (aWISH) aims to improve animal welfare through automated animal welfare indicator (AWI) monitoring at the slaughterhouse.**

**Within this project, “Best Practice Guides” will be developed that provide practical recommendations to improve animal welfare on farm, during transport and at the slaughterhouse.**

**Based on a survey with experts from research and academia, this survey now aims to identify the opinion of farmers regarding those best practices.**

**There are two parts in this survey:
1. Information on the participants’ background
2. Evaluation of Best Practices
It will take about 10 minutes to complete.**

**All data collected will be treated as confidential and will not be disclosed to any third parties.**

**Thank you for participating!**

**If you have any questions regarding the survey, please do not hesitate to contact us via email:** [**maite.marie.jachens@tiho-hannover.de**](mailto:maite.marie.jachens@tiho-hannover.de)

**TiHo (aWISH partner)**

**Part 1: Participant’s background**

1) **What is your background?**
   - Self-employed farmer
   - Employed farmer
   - Agricultural Service Provider
   - Other (please specify)
2) **How many years have you been working in this field?**
3) **What is your highest level of education?**

- No qualification
- Non-academic qualifications (school diploma/vocational training)
- Meister/foreman
- University degree (Bachelor/Master/PhD)
- PhD

4) **Do you work with other animals apart from pigs?**

- Broilers
- Laying hens
- Cattle
- Other (please specify)
- No

5) **How old are you?**
6) **What is your gender identity?**

- Male
- Female
- Non-binary
- Prefer not to say

7) **Which country do you currently work in?**

**Part 2: Evaluation of Best Practices**

1) **Please indicate how often you have experienced the following animal welfare issues in pigs: *(often, sometimes, rarely, never)***

- Tail biting/ear biting
- Skin lesions
- Bursitis

2) **Below you will find a list of measures to prevent or reduce the aforementioned animal welfare issues. Please rate the suitability of these measures on a scale from 0 to 100% (0% = unsuitable; 100% = very suitable).
   If you are unsure about a measure and/or would prefer not to rate it, please use the “reset” button.**

**Tail lesions/Ear lesions, Skin lesions:**

- Organic manipulable material on farm except straw (wood, sisal ropes, pressed molasses, etc)
- Non-organic manipulable material on farm (metal chains, chewable plastic material, etc)
- Provision of straw or hay as enrichment (in racks, feeders, etc.)
- Provision of straw as bedding on farm
- Regular refilling of manipulable or bedding material on farm (so it doesn’t run out)
- Stocking density on farm according to EU legislation
- Lowering stocking density on farm below EU legislation / more space allowance per pig
- Very small group size on farm (similar to litter size: 10-16 pigs)
- Small group size on farm (< 40 pigs per group)
- Bigger group size on farm (> 40 pigs per group)
- Keeping littermates together from birth to the fattening/finishing period (no mixing)
- As little regrouping / mixing of animals on farm as possible
- Structuring of the pen (lying, feeding, dunging area)
- Pens with outdoor area
- Good health status
- Good barn climate / air quality (e.g. little ammonia, optimal air flow, etc)
- No storage of manure in the stable (under the slatted floor)
- Good feed quality (e.g. no mycotoxins, balanced)
- Enough feeding space per pig (preferably 1:1)
- Good feeding management (e.g. ad libitum, short feeding intervals)
- Genetic aspects (genetics that are preferred in countries without tail docking)
- Gentle animal handling

**Bursitis:**

- Bedding (e.g. straw)
- Soft or soil floors, no slats
- When using slats: plastic rather than concrete or metal ones
- Non-slatted concrete floor
- Rubber floor mats
- Good floor condition (e.g no breakage, non-slippery)

**Thank you for participating in this survey!**

**If you have any questions or suggestions, please do not hesitate to contact us via email:** [**maite.marie.jachens@tiho-hannover.de**](mailto:maite.marie.jachens@tiho-hannover.de)

**TiHo (aWISH partner)**

**Privacy policy and declaration of consent**

**1. Purpose of processing**

We collect and process the personal data to be used for participation in the above survey. The purpose of this survey is to identify Best Practices regarding animal welfare of fattening pigs.

The results of the survey will be used by the Institute of Animal Hygiene, Animal Welfare and Farm Animal Behaviour (ITTN) for research, presentation and publication purposes; if personal information is available, it will be anonymized beforehand, Art. 6 I 1 lit. e in conjunction with 89 GDPR, § 3 I 1 No. 1 NHG, § 13 NDSG.

**2. Legal basis of data processing**

Participation in the survey is voluntary. When completing the survey, your IP address will be transmitted to the clinic server and stored there - at least temporarily. By participating in the survey, you agree to this storage in accordance with Art. 6 I 1 lit. a GDPR. No other personal data will be processed. All data will be treated securely and confidentially. The survey was designed using software provided for this purpose by LimeSurvey on the clinic server. In addition, data protection notices from LimeSurvey can be viewed online: <https://www.limesurvey.org/de/richtlinien/datenschutzrichtlinie>.

The possibility of use for teaching, research, lecture and publication purposes results from Art. 6 I 1 lit. e GDPR in conjunction with. § 13 NDSG. The possibility to process data for evaluation purposes can be derived from Art. 6 I 1 lit. e GDPR in conjunction with. § 5 NHG.

**3. Duration of processing**

The personal data will be stored for the duration of the survey and the research project beyond. Deletion takes place automatically, at the latest 6 months after the end of the project. Legal storage obligations remain unaffected by this.

**4. Rights of the persons concerned**

You have a right of access to the personal data concerning you and, if applicable, to correction, deletion or restriction of the processing of this data and a right to object to the processing as well as a possible right to data portability vis-à-vis the institution named below. You may withdraw your consent, if any, at any time with effect for the future, Art. 7 III GDPR. In addition, you have a right to submit complaints to the supervisory authority if you believe that the processing of personal data concerning you is in conflict with the law. The competent supervisory authority is the State Commissioner for Data Protection of Lower Saxony.

| Supervisor | Contact person | Data protection officer |
| --- | --- | --- |
| Institute of Animal Hygiene, Animal Welfare and Farm Animal Behaviour (ITTN)  University of Veterinary Medicine, Foundation (TIHO) | Institute of Animal Hygiene, Animal Welfare and Farm Animal Behaviour (ITTN)  University of Veterinary Medicine, Foundation (TIHO) | University of Veterinary Medicine, Foundation (TIHO) |
| Michaela Fels | Maite Jachens | Wolfgang Rottwinkel |
| Bischofsholer Damm 15 (Gebäude 116)  30173 Hannover | Bischofsholer Damm 15 (Gebäude 116)  30173 Hannover | Bünteweg 2, 30559 Hannover |
| Tel.: +49 511 953 8954 | Tel.: +49 511 953 8966 | Tel.: +49 511 953 8015 |
|  |  | Fax: 0511 953 828015 |
| E-Mail: michaela.fels@tiho-hannover.de | E-Mail: maite.marie.jachens@tiho-hannover.de | E-Mail: datenschutz@tiho-hannover.  de |
