# Supplementary material for: It’s all a matter of perspective—a comparison of scientific experts’ vs. farmers’ opinions on Best Practices for pig welfare
Source: Front Vet Sci. 2026 Sep 23;13:1905492. doi: 10.3389/fvets.2026.1905492 (PMC13643911; doi:10.3389/fvets.2026.1905492)
Supplement: Supplementary file 2 [file Table_2.DOCX]

Survey template for scientific experts

**Identifying Best Practices to improve Animal Welfare for fattening pigs**

**This survey is part of the EU project “Animal Welfare Indicators at the Slaughterhouse” (aWISH), Grant Agreement ID 101060818.**

**For more information on aWISH see our project website:** <https://www.awish-project.eu/>

**Within this project, Best Practice Guides will be developed that provide practical recommendations to improve animal welfare for fattening pigs along the production chain.
The aim of this survey is to gather expert knowledge regarding those best practices.**

**There are two parts in this survey:
1. Information on the participants’ background
2. Evaluation of Best Practices regarding Animal Welfare Indicators (AWI)**

**Your responses are completely anonymous. The information collected will be used in the development of the afore mentioned Best Practice Guides.**

**We really appreciate your input!**

**If you have any questions regarding the survey, please do not hesitate to contact us via email:** [maite.marie.jachens@tiho-hannover.de](mailto:maite.marie.jachens@tiho-hannover.de)

**TiHo (aWISH partner)**

**Part 1: Participant’s background**

1) **What is your background?**
   - Research/Academia
   - Technology provider
   - Consulting
   - Industry
   - Slaughterhouse
   - Other (please specify)
2) **Are you part of aWISH?**

- Yes, I am working for one of the aWISH partners.
- Yes, I am on the expert panel.
- No, I am not part of the aWISH project.

3) **Please indicate how many years of experience you have in the field listed below.**

- Animal behaviour/Animal welfare
- Animal production/Agriculture
- Veterinary medicine
- Food inspection/Meat hygiene

4) **Do you have experience in any other relevant field that is not mentioned above? If so, please specify:**
5) **Which country do you currently work in?**

**Part 2: Evaluation of Best Practices regarding Animal Welfare Indicators (AWI)**

1) **Are you scientifically experienced with pigs?**

- Yes
- No

2) **A literature research was conducted to identify Best Practices on farm, on transport and at the slaughterhouse for specific animal welfare indicators (AWI).
   Below you will find a list of measures for each AWI. Please rate the suitability of these measures on a scale from 0 to 100%.
   If you are unsure about a measure and/or would prefer not to rate it, please use the “reset” button.**

**Tail lesions/Ear lesions:**

- Organic manipulable material on farm except straw (wood, sisal ropes, pressed molasses, etc)
- Non-organic manipulable material on farm (metal chains, chewable plastic material, etc)
- Provision of straw or hay as enrichment (in racks, feeders, etc.)
- Provision of straw as bedding on farm
- Regular refilling of manipulable or bedding material on farm (so it doesn’t run out)
- Stocking density on farm according to EU legislation
- Lowering stocking density on farm below EU legislation / more space allowance per pig
- Very small group size on farm (similar to litter size: 10-16 pigs)
- Small group size on farm (< 40 pigs per group)
- Bigger group size on farm (> 40 pigs per group)
- Keeping littermates together from birth to the fattening/finishing period (no mixing)
- As little regrouping / mixing of animals on farm as possible
- Structuring of the pen (lying, feeding, dunging area)
- Pens with outdoor area
- Good health status
- Good barn climate / air quality (e.g. little ammonia, optimal air flow, etc)
- No storage of manure in the stable (under the slatted floor)
- Good feed quality (e.g. no mycotoxins, balanced)
- Enough feeding space per pig (preferably 1:1)
- Good feeding management (e.g. ad libitum, short feeding intervals)
- Genetic aspects (genetics that are preferred in countries without tail docking)

**Skin lesions:**

- As little regrouping / mixing of animals on farm as possible
- Little regrouping / mixing of animals for transport
- Little regrouping / mixing of animals in lairage area at the slaughterhouse
- Keeping littermates together from birth to the fattening/finishing period (no mixing)
- Organic manipulable material on farm except straw (wood, sisal ropes, pressed molasses, etc)
- Non-organic manipulable material on farm (metal chains, chewable plastic material, etc)
- Provision of straw or hay on farm as enrichment (in racks, feeders, etc.)
- Provision of straw as bedding on farm
- Regular refilling of manipulable or bedding material on farm (no running out)
- Stocking density on farm according to EU legislation
- Lowering stocking density on farm below EU legislation / more space allowance per pig
- Very small group size on farm (similar to litter size: 10-16 pigs)
- Small group size on farm (< 40 pigs per group)
- Bigger group size on farm (> 40 pigs per group)
- Structuring of the pen (lying, feeding, dunging area)
- Short lairage stays at the slaughterhouse
- Stocking density on transport according to EU legislation
- Short transport duration (< 9 h)
- Gentle animal handling on farm
- Gentle animal handling on transport
- Gentle animal handling at the slaughterhouse

**Bursitis:**

- Bedding (e.g. straw)
- Soft or soil floors, no slats
- When using slats: plastic rather than concrete or metal ones
- Non-slatted concrete floor
- Rubber floor mats
- Good floor condition (e.g no breakage, non-slippery)

**Thank you for your participation! If you have any questions regarding this survey, please do not hesitate to contact us via email:** [maite.marie.jachens@tiho-hannover.de](mailto:maite.marie.jachens@tiho-hannover.de)**.**

**TiHo (aWISH partner)**

**Privacy policy and declaration of consent**

**1. Purpose of processing**

We collect and process the personal data to be used for participation in the above survey. The purpose of this survey is to identify Best Practices regarding animal welfare of fattening pigs.

The results of the survey will be used by the Institute of Animal Hygiene, Animal Welfare and Farm Animal Behaviour (ITTN) for research, presentation and publication purposes; if personal information is available, it will be anonymized beforehand, Art. 6 I 1 lit. e in conjunction with 89 GDPR, § 3 I 1 No. 1 NHG, § 13 NDSG.

**2. Legal basis of data processing**

Participation in the survey is voluntary. When completing the survey, your IP address will be transmitted to the clinic server and stored there - at least temporarily. By participating in the survey, you agree to this storage in accordance with Art. 6 I 1 lit. a GDPR. No other personal data will be processed. All data will be treated securely and confidentially. The survey was designed using software provided for this purpose by LimeSurvey on the clinic server. In addition, data protection notices from LimeSurvey can be viewed online: <https://www.limesurvey.org/de/richtlinien/datenschutzrichtlinie>.

The possibility of use for teaching, research, lecture and publication purposes results from Art. 6 I 1 lit. e GDPR in conjunction with. § 13 NDSG. The possibility to process data for evaluation purposes can be derived from Art. 6 I 1 lit. e GDPR in conjunction with. § 5 NHG.

**3. Duration of processing**

The personal data will be stored for the duration of the survey and the research project beyond. Deletion takes place automatically, at the latest 6 months after the end of the project. Legal storage obligations remain unaffected by this.

**4. Rights of the persons concerned**

You have a right of access to the personal data concerning you and, if applicable, to correction, deletion or restriction of the processing of this data and a right to object to the processing as well as a possible right to data portability vis-à-vis the institution named below. You may withdraw your consent, if any, at any time with effect for the future, Art. 7 III GDPR. In addition, you have a right to submit complaints to the supervisory authority if you believe that the processing of personal data concerning you is in conflict with the law. The competent supervisory authority is the State Commissioner for Data Protection of Lower Saxony.

| Supervisor | Contact person | Data protection officer |
| --- | --- | --- |
| Institute of Animal Hygiene, Animal Welfare and Farm Animal Behaviour (ITTN)  University of Veterinary Medicine, Foundation (TIHO) | Institute of Animal Hygiene, Animal Welfare and Farm Animal Behaviour (ITTN)  University of Veterinary Medicine, Foundation (TIHO) | University of Veterinary Medicine, Foundation (TIHO) |
| Michaela Fels | Maite Jachens | Wolfgang Rottwinkel |
| Bischofsholer Damm 15 (Gebäude 116)  30173 Hannover | Bischofsholer Damm 15 (Gebäude 116)  30173 Hannover | Bünteweg 2, 30559 Hannover |
| Tel.: +49 511 953 8954 | Tel.: +49 511 953 8966 | Tel.: +49 511 953 8015 |
|  |  | Fax: 0511 953 828015 |
| E-Mail: michaela.fels@tiho-hannover.de | E-Mail: maite.marie.jachens@tiho-hannover.de | E-Mail: datenschutz@tiho-hannover.  de |
